# Telepresence Robots at the Urology and Emergency Department: A Pilot Study Assessing Patients' and Healthcare Workers' Satisfaction

**DOI:** 10.1155/2022/8787882

**Published:** 2022-03-15

**Authors:** Jens Laigaard, Trine Ungermann Fredskild, Grzegorz Lukasz Fojecki

**Affiliations:** ^1^Department of Urology, Hospital of Southern Jutland, 6200 Aabenraa, Denmark; ^2^The Learning and Research Department, Hospital of Southern Jutland, University Hospital of Southern Denmark, 6200 Aabenraa, Denmark

## Abstract

COVID-19 intensified interest in telemedicine, yet no study has evaluated the use of a telepresence robot on unselected urological patients. Therefore, we performed a survey study of patients, bedside caregivers and urologists, investigating the satisfaction and applicability of a telepresence robot (Beam Pro, Suitable Technologies, USA) at the urology ward and emergency department. The primary outcome was the number of patient encounters solved without the urologist's physical presence. Between March 2021 and May 2021, patients, caregivers, and urologists filled in 42, 35, and 54 questionnaires, respectively. Most patients were male (79%), with a mean age of 64 (SD ± 17). Two of the department's ten urologists participated. The urologists responded that physical examination was required in 7 (13%) encounters. The caregivers would have preferred the urologist physically present in 11 (31%) cases. Most patients (71%) “agreed” or “strongly agreed” that they were willing to be attended by a telepresence robot at future evaluations and generally, patients gave high satisfaction scores. Though implementation among the department's urologists was a major challenge, participating urologists reported that physical presence could be avoided in 87% of the patient encounters. Studies of patient-reported outcome measures comparing telemedical and physical patient encounters are needed.

## 1. Introduction

Telemedicine has gained huge momentum in the COVID-19 era because it reduces physical contact and thus the risk of virus transmission [1, 2]. Moreover, telemedicine has obvious logistic benefits. It reduces the carbon footprint and supports equality in healthcare [3]. Therefore, it has been studied in multiple urological patient groups, mainly for follow-up visits and in ambulatory settings [2]. Studies have generally shown high patient satisfaction but have been criticized for including a very select group of patients, limiting generalizability [2].

Telemedicine refers to any delivery of remote medical care (i.e., using telecomunication). A branch of telemedicine, telepresence, refers to technologies that allow the user to feel as if they are present and perform tasks in a remote location, for example, using a telepresence robot. While a few studies have shown high accuracy results with the use of telepresence robots during postoperative rounds in the urology departments [4–6], and for selected, simple diagnoses in the emergency department [7, 8], no trials have evaluated the use of telepresence robots in unselected urological patients.

**Figure 1:**
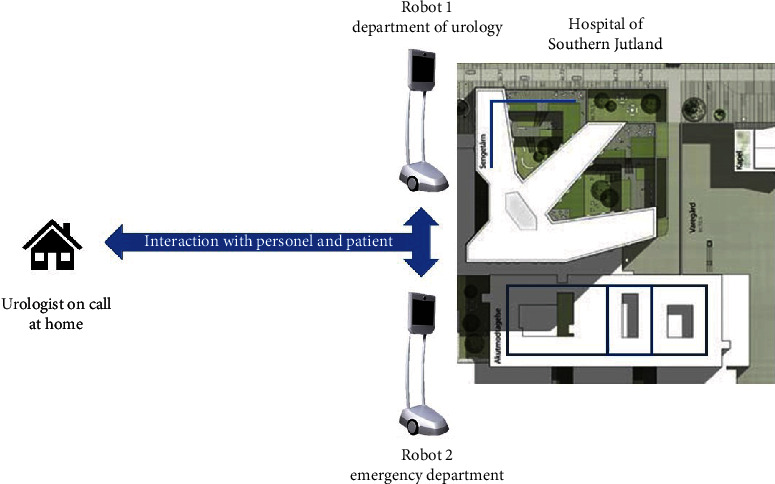
Geographical system architecture. Two different telepresence robots were used in this study: one located at the urology ward and one located at the emergency department.

At the Hospital of Southern Jutland, the Department of Urology introduced two telepresence robots (Beam Pro, Suitable Technologies, USA): one to assist during ward rounds and one to treat acute patients at the emergency department (Figure 1).

The robots are equipped with a battery, wheels, and two cameras to allow safe navigation around the department from a remote location. In addition, a microphone, speakers, and a 14 inch screen allow staff and patients to interact [9]. Combined with a peer-to-peer latency of ≈10 milliseconds, this provides a sense of presence lacking only the tactile component.

We aimed to assess the applicability and satisfaction with telepresence robots for emergency care and rounds at the urology department among patients and healthcare workers.

## 2. Materials and Methods

This prospective qualitative study was conducted following the principles of the Declaration of Helsinki and followed the predefined protocol (available upon request), approved by the local scientific committee. In Denmark, survey studies are not reviewed by the central ethics committee. Data were stored according to national data protection legislation. Before consenting, all participants were verbally informed that participation was voluntary, and that quality of care would not differ if they chose not to participate.

### 2.1. Telepresence Robots

The telepresence robots can be utilized from any computer with Internet access and the Beam App installed using personal login credentials [9]. In order to log in and establish a Beam session, this port must be open on the computer's network firewall. The TCP port 443 is used for HTTPS traffic, and UDP ports 6868-6871 are used for relayed or P2P audio/video traffic, where call establishment may be assisted by STUN. At the Hospital of Southern Jutland, our local IT technicians opened the network firewall by creating a network for the Beam robots only.

The telepresence robots utilize dual-band wireless radios and patented WiFi-LTE (4G) roaming algorithms, which are compatible with IEEE 802.11 a/b/g/n WiFi signals. Communications within the Beam system are normally transmitted via direct peer-to-peer UDP connections, utilizing industry-standard encryption (AES-256) and authentication (HMAC-SHA1) technology, along with random number-generated keys, to optimize privacy and security. Since encryption/decryption occur at call endpoints, security is maintained even when inability to establish a direct connection requires use of Beam public relays. Independent security audits were performed by Optiv (formerly Accuvant) and Gotham.

### 2.2. Inclusion and Exclusion Criteria

All urologists and caregivers working at the department of urology or the emergency department were eligible.

Eligible patients were all adults (>18 years) admitted to the emergency or urology department with a suspected urological problem, who could read and understand Danish and give informed consent to participate.

### 2.3. Study Design

**Figure 2:**
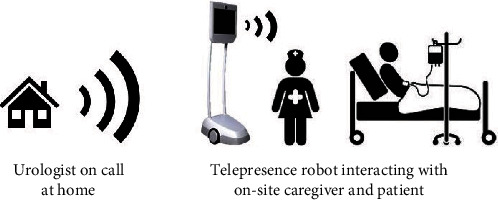
Communication via the telepresence robot. Using the telepresence robot, the urologists can move around and interact with patients and on-site caregivers with audio and video.

The study was conducted at the Hospital of Southern Jutland, Aabenraa, Denmark, a hospital that provides urological care for 250,000 patients in a radius of approximately 50 km. At the urologists' discretion, patients may be seen using the telepresence robot together with an on-site caregiver (Figure 2).

We designed a pragmatic survey study, with optional, anonymous participation for physicians, caregivers, and patients, immediately after the telemedicine encounter.

### 2.4. Outcomes

The primary outcome was the number of encounters requiring a physical examination by the urologist.

Secondary outcomes included user satisfaction and the ability to achieve a safe, natural conversation.

### 2.5. Data Collection

The on-call urologist used a laptop to access relevant electronic health records and had access to software that allows a safe, encrypted connection with the telepresence robot [9]. The on-call urologists accessed the telepresence robot either from an on-site hospital office or from home. They attended rounds and consultations at the emergency department without any physical contact. Whenever a medical problem could not be solved using the robot, the urologist attended the patient physically. After an encounter ended, the urologist informed the patient and the on-site caregiver about the possibility to anonymously and voluntarily evaluate the encounter. Printed copies of the questionnaire were available from containers attached to the robot and were later entered into online forms by the authors (JL and GF). Concurrently, the operator answered the questionnaire using an online form. As a result, one encounter could potentially result in three questionnaire responses. The questionnaires are available in Appendix 1 (Danish) and Appendix 2 (English translation).

### 2.6. Statistics

No sample size calculation was performed as no hypothesis testing was planned. Data was organized using Excel version 16.0 (Microsoft, Redmond, WA, USA) and analyzed using *R* i386 version 3.6 (https://www.r-project.org/). Data was presented as mean (±SD), median (interquartile range (IQR)), or absolute numbers and percentages as appropriate.

## 3. Results

### 3.1. Participants

Between March 2021 and May 2021, patients, caregivers, and urologists filled in 42, 35, and 54 questionnaires, respectively. Two (20%) of the department's ten urologists with access to the telepresence robot filled in questionnaires. Among the patients, 79% were men. The mean age of the patients was 64 (SD ± 17). Further details are presented in Table 1.

The most frequent reasons for being seen by an urologist were rounds (12 (22%)), urinary tract infections (9 (17%)), discharge (5 (9%)), urolithiasis (5 (9%)), and hematuria (4 (7%)). A full list of reasons can be seen in Appendix 3.

### 3.2. Primary Outcome

The urologists assessed that physical examinations were required in four (20%) of the 20 encounters in the emergency department and in three (9%) of the 34 encounters at the urology department. Of these, three (6%) were patients requiring bedside ultrasound examination, and two (4%) were mentally disabled patients requiring the urologist's physical presence.

### 3.3. Secondary Outcomes

Despite rating the image quality as “average” (Table 2), the urologists were able to assess the clinical status (median 5 (IQR 5-5)) and face expression (median 5 (IQR 5-5)) of the patients, on a 1-5 scale where 1 =  ^“^not at all^”^ and 5 =  ^“^like being there in person.^”^

The caregivers would have preferred the physical presence of a urologist in 11 (31%) cases. However, despite this response, the caregivers generally gave high satisfaction scores to the use of the telepresence robot (Table 2).

**Figure 3:**
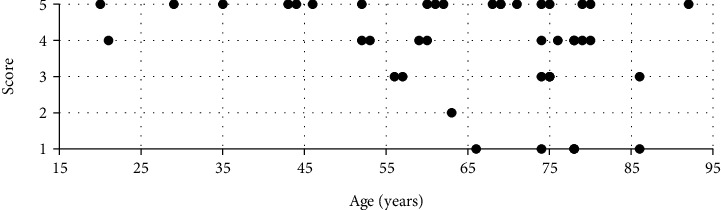
Scatterplot of patients' attitude against the telepresence robot divided on age (*n* = 42). Axes are patients' age and willingness to be seen by the telepresence robot at a future visit on a 1-5 scale (where 1 = ^“^strongly disagree^”^ and 5 = ^“^strongly agree^”^).

Most patients “agreed” or “strongly agreed” that in the future, they would like to encounter the robot again, though six patients (14%) “disagreed” or “strongly disagreed” (Figure 3). Generally, the patients rated the image and sound quality as good, and most felt safe with the conversation (Table 2).

## 4. Discussion

This survey was conveyed as a pilot study to evaluate if a telepresence robot could effectively be used in an in-patient setting. According to the urologists' who participated, physical presence was not necessary in 87% of the encounters. Furthermore, patients and caregivers generally responded favorably to satisfaction measures, and patients expressed willingness to use a telepresence robot in future examinations.

The participants' ages were well dispersed because of the unselected patient population. More men than women participated in this study, partly because many postoperative examinations (rounds, discharge) at the ward involved prostate surgery.

To our knowledge, this is the first study evaluating the applicability of telemedicine in unselected urological patients. Previous studies on telemedicine in urology have mostly reported encouraging results [10–14]. Notably, many ambulatory visits can be conducted satisfactorily using videoconferencing equipment [10, 11, 14, 15]. Likewise, studies on the use of telepresence for rounds, telerounding, at urology departments have generally yielded positive results by including a highly selected patient group [4–6, 16]. Two randomized controlled trials by Ellison et al. showed high satisfaction and no clinical downsides with the use of a telepresence robot for ward rounds after selected urologic procedures [4, 5]. In contrast, Oh et al. reported markedly lower satisfaction with telerounding compared to conventional rounding after urological surgery [6]. Our study is unique because we included all hospitalized patients and similar to the Californian study [4, 5], we found high end-user satisfaction with telemedicine.

Studies on the use of telemedicine in emergency care settings are relatively sparse [7, 8, 17]. Two randomized controlled trials by Brennan et al. reported a similar study design to ours but only included a limited number of medical conditions (e.g., abrasions, insect bites, and simple cystitis) [7, 8]. In both trials, participants in the intervention group were evaluated using a telepresence robot and gave satisfaction scores similar to participants in the control group and reported no adverse clinical effects. However, these trials are more than 20 years old, and electronic equipment has substantially improved [7, 8]. We found a high degree of satisfaction among unselected patients; although, we have no comparator group.

Another study, Sherwood et al. retrospectively investigated phone and video calls to inmates with unselected urological complaints [17]. Initially, a primary care provider had seen the inmate patients, and the subsequent telemedical visit triaged patients into those who needed or did not need a physical examination. The telepresence consultation correctly identified a specific diagnosis in 90% of the cases, and the authors estimated that 52% of the complaints could be solved safely with telemedicine alone [17].

In our opinion, the user-friendly software and the ability to move independently make communication telepresence robots superior comparing to other solutions (e.g., tablets or telephone).

### 4.1. Limitations

In our study, urologists could solve 87% of the patient encounters using a telepresence robot and help from an on-site caregiver. However, the urologists selected which patients they would see using the telepresence robot and which they would be physically present to examinate. Thereby, patients who were deemed complex a priori or in need of an acute intervention may have been seen physically right away. On the other hand, the referral complaint frequently does not concur with the actual complaint. Also, patients and caregivers did not choose whether to meet a telepresence robot.

Because only two of the department's urologists filled in questionnaires, the generalizability of the operator responses is limited. The low participation rate among urologists at our department was caused mainly by a lack of evidence and clear guidelines on how and when to apply a telepresence robot.

Because we had no randomization, comparison group, or clinical outcomes, this study cannot provide solid evidence for the usability of a telepresence robot. However, the high degree of user satisfaction and other patient-reported outcome measures support applying this technology in clinical settings.

Lastly, the telepresence robots are only applicable at departments where doctors are on call from home or departments with patients at multiple geographical locations, which limit the generalizability of our results.

## 5. Conclusion

We conducted a survey study of patients' and healthcare professionals' satisfaction with a telepresence robot at the urology and emergency department. Though implementation among the department's urologists was a major challenge, participating urologists reported that physical presence was not necessary for 87% of the patient encounters. Patients and healthcare professionals were generally satisfied with the telemedicine solution. Studies comparing patient-reported outcome measures between telemedical and physical patient encounters are needed.

## Figures and Tables

**Table 1:** Participant demographics.

	Patients (*n* = 42)
Emergency/urology department	15/27
Age (years)	64 (SD ± 17)
Gender (women/men)	9/33

	Assistants (*n* = 35)
Emergency/urology department	18/17
Position	
Nurse	20 (57%)
Medical student	7 (20%)
Medical intern	2 (6%)
Other healthcare professional	6 (17%)

Legend: numbers indicate filled in questionnaires.

**Table 2:** Questionnaire responses.

	Patients (*n* = 42)	Assistants (*n* = 35)	Urologists (*n* = 54)
From 1-5 where 1 = ^“^very bad^”^ and 5 = ^“^very good^”^			
How do you rate the image quality?	5 (4-5)	5 (4-5)	3 (3-3)
How do you rate the sound quality?	5 (4-5)	5 (4-5)	5 (5-5)
How do you rate the robot's mobility?	—	—	5 (5-5)
From 1-5 where 1 = ^“^strongly disagree^”^ and 5 = ^“^strongly agree^”^			
We succeeded in having a natural conversation	4 (4-5)	5 (4-5)	5 (5-5)
I felt safe through the conversation	5 (4-5)	5 (5-5)	—
I was able to convey the patient's treatment plan	—	5 (5-5)	5 (5-5)
I was able to assess the color of the urine	—	—	5 (5-5)
			NR: 29
			NA: 1
I was able to assess the ultrasound image	—	—	5 (4-5)
			NR: 45

Numbers are median (IQR). NR: not relevant; NA: not available.

## Data Availability

The questionnaire data used to support the findings of this study are available from the corresponding author (GF) upon request.

## Supplementary Materials

Supplementary Materials Appendices are supplied in the supplementary material consisting of (1) the original questionnaires in Danish, (2) the questionnaires translated into English, and (3) a list of the reasons for being seen by an urologist using the robot in the study period.
