# Supplementary material for: Telepresence Robots at the Urology and Emergency Department: A Pilot Study Assessing Patients' and Healthcare Workers' Satisfaction
Source: Int J Telemed Appl. 2022 Mar 15;2022:8787882. doi: 10.1155/2022/8787882 (PMC8941569; doi:10.1155/2022/8787882)
Supplement: Supplementary Materials — Appendices are supplied in the supplementary material consisting of (1) the original questionnaires in Danish, (2) the questionnaires translated into English, and (3) a list of the reasons for being seen by an urologist using the robot in the study period. [file 8787882.f1.docx]

Supplementary material:
*Telepresence Robots at the Urology and Emergency Department. A pilot study assessing Patients’ and Health-care Workers’ satisfaction.*

Jens Laigaard^a^, (ORCID 0000-0001-5297-3769)
Trine Ungermann Fredskild^b^ (ORCID 0000-0002-8146-6501)
Grzegorz Lukasz Fojecki^a^* (ORCID 0000-0001-5349-1585)

^a^Department of Urology, Sygehus Sønderjylland, Aabenraa, Denmark;

^b^The Learning and Research department, Hospital Sønderjylland, University Hospital of Southern Denmark

*Corresponding author. [fgrzech@wp.pl](mailto:fgrzech@wp.pl). Hospital of Southern Jutland, Aabenraa, Kresten Philipsens vej 15, 6200 Aabenraa, DK

Appendix 1: Questionnaires in Danish

**Udfyldes af patienten**

**KØN: ALDER:**

**_________________ ______________________**

**På en skala hvor 1 er ’Meget dårlig’ – og 5 er ’Meget god’**

| Hvordan vurderer du billedkvaliteten? | 1 | 2 | 3 | 4 | 5 |
| --- | --- | --- | --- | --- | --- |
| Hvordan vurderer du lydkvaliteten? | 1 | 2 | 3 | 4 | 5 |

**Hvor enig er du i udsagnene, på en skala hvor 1 er ’helt uenig’ – og 5 er ’helt enig’**

| Det lykkedes at have en naturlig samtale | 1 | 2 | 3 | 4 | 5 |
| --- | --- | --- | --- | --- | --- |
| Jeg var tryg ved samtalen | 1 | 2 | 3 | 4 | 5 |
| Jeg kunne forestille mig at blive vurderet gennem robotten ved et fremtidigt besøg | 1 | 2 | 3 | 4 | 5 |

*Fundne spørgeskemaer bedes returneret til*

*Grzegorz Lukasz Fojecki, læge ph.d.*[*Grzegorz.Lukasz.Fojecki@rsyd.dk*](mailto:Grzegorz.Lukasz.Fojecki@rsyd.dk) *Team Station 1*

**Udfyldes af robotoperatøren**

**DINE INITIALER**

**_____________________________**

**Diagnose/problem:**

**________________________________________________________________________________________________**

**Sted: FAM Afd.**

**På en skala hvor 1 er ’Meget dårlig’ – og 5 er ’Meget god’**

| Hvordan vurderer du billedkvaliteten? | 1 | 2 | 3 | 4 | 5 |
| --- | --- | --- | --- | --- | --- |
| Hvordan vurderer du lydkvaliteten? | 1 | 2 | 3 | 4 | 5 |
| Hvordan vurderer du robottens evne til at bevæge sig omkring? | 1 | 2 | 3 | 4 | 5 |

**Hvor enig er du i udsagnene, på en skala hvor 1 er ’helt uenig’ – og 5 er ’helt enig’**

| Det var muligt at vurdere patientens kliniske tilstand | 1 | 2 | 3 | 4 | 5 |
| --- | --- | --- | --- | --- | --- |
| Det var muligt at aflæse patientens ansigtsudtryk | 1 | 2 | 3 | 4 | 5 |
| Det lykkedes at have en naturlig samtale | 1 | 2 | 3 | 4 | 5 |
| Det var muligt for at formidle en behandlingsplan | 1 | 2 | 3 | 4 | 5 |
| Det var muligt at vurdere urinens farve (Hvis relevant) | 1 | 2 | 3 | 4 | 5 |
| Det var muligt at aflæse ultralydsscanneren (Hvis relevant) | 1 | 2 | 3 | 4 | 5 |

**Var det nødvendigt at tilse patienten fysisk?**

Ja Nej

**Udfyldes af sygeplejerske/forvagt**

**STILLING:**

**__________________________________________________**

**Diagnose/problem:**

**________________________________________________________________________________________________**

**På en skala hvor 1 er ’Meget dårlig’ – og 5 er ’Meget god’**

| Hvordan vurderer du billedkvaliteten? | 1 | 2 | 3 | 4 | 5 |
| --- | --- | --- | --- | --- | --- |
| Hvordan vurderer du lydkvaliteten? | 1 | 2 | 3 | 4 | 5 |

**Hvor enig er du i udsagnene, på en skala hvor 1 er ’helt uenig’ – og 5 er ’helt enig’**

| Det lykkedes at have en naturlig samtale | 1 | 2 | 3 | 4 | 5 |
| --- | --- | --- | --- | --- | --- |
| Jeg var tryg ved samtalen | 1 | 2 | 3 | 4 | 5 |
| Det var muligt at formidle lægens behandlingsplan | 1 | 2 | 3 | 4 | 5 |

**Ville du foretrække at lægen havde været til stede fysisk?**

Ja Nej

*Fundne spørgeskemaer bedes returneret til*

*Grzegorz Lukasz Fojecki, læge ph.d.*[*Grzegorz.Lukasz.Fojecki@rsyd.dk*](mailto:Grzegorz.Lukasz.Fojecki@rsyd.dk) *Team Station 1*

Appendix 2: Questionnaires in English

**Filled in by the patient**

**Gender: Age:**

**_________________ ______________________**

**On a scale where 1 is ’very bad’ – and 5 is ‘very good’**

| How do you rate the image quality? | 1 | 2 | 3 | 4 | 5 |
| --- | --- | --- | --- | --- | --- |
| How do you rate the sound quality? | 1 | 2 | 3 | 4 | 5 |

**Please rate the following statements on a scale from 1= ‘strongly disagree’ – and 5= ‘strongly agree’**

| We succeeded in having a natural conversation | 1 | 2 | 3 | 4 | 5 |
| --- | --- | --- | --- | --- | --- |
| I felt safe through the conversation | 1 | 2 | 3 | 4 | 5 |
| I do imagine being evaluated using the robot at a future visit | 1 | 2 | 3 | 4 | 5 |

*Please return questionnaires to*

*Grzegorz Lukasz Fojecki, MD, ph.d.*[*Grzegorz.Lukasz.Fojecki@rsyd.dk*](mailto:Grzegorz.Lukasz.Fojecki@rsyd.dk) *Team Station 1*

**Filled in by the robot operator**

**Your initials**

**_____________________________**

**Diagnosis/problem:**

**________________________________________________________________________________________________**

**Location: ED Ward**

**On a scale where 1 is ’very bad’ – and 5 is ‘very good’**

| How do you rate the image quality? | 1 | 2 | 3 | 4 | 5 |
| --- | --- | --- | --- | --- | --- |
| How do you rate the sound quality? | 1 | 2 | 3 | 4 | 5 |
| How do you rate the robot’s ability to move around? | 1 | 2 | 3 | 4 | 5 |

**Please rate the following statements on a scale from 1= ‘strongly disagree’ – and 5= ‘strongly agree’**

| I was able to evaluate the patient’s clinical status | 1 | 2 | 3 | 4 | 5 |
| --- | --- | --- | --- | --- | --- |
| I was able to read the patient’s face expressions | 1 | 2 | 3 | 4 | 5 |
| We succeeded in having a natural conversation | 1 | 2 | 3 | 4 | 5 |
| I was able to convey the patient’s treatment plan | 1 | 2 | 3 | 4 | 5 |
| I was able to assess the colour of the urine (if relevant) | 1 | 2 | 3 | 4 | 5 |
| I was able to assess the ultrasound image (if relevant) | 1 | 2 | 3 | 4 | 5 |

**Was it necessary to see the patient in person?**

Yes No

**Filled in by assistant**

**Position:**

**__________________________________________________**

**Diagnosis/problem:**

**________________________________________________________________________________________________**

**On a scale where 1 is ’very bad’ – and 5 is ‘very good’**

| How do you rate the image quality? | 1 | 2 | 3 | 4 | 5 |
| --- | --- | --- | --- | --- | --- |
| How do you rate the sound quality? | 1 | 2 | 3 | 4 | 5 |

**Please rate the following statements on a scale from 1= ‘strongly disagree’ – and 5= ‘strongly agree’**

| We succeeded in having a natural conversation | 1 | 2 | 3 | 4 | 5 |
| --- | --- | --- | --- | --- | --- |
| I felt safe through the conversation | 1 | 2 | 3 | 4 | 5 |
| I was able to convey the patient’s treatment plan | 1 | 2 | 3 | 4 | 5 |

**Would you prefer the urologist being there in person?**

Yes No

*Please return questionnaires to*

*Grzegorz Lukasz Fojecki, MD, ph.d.*[*Grzegorz.Lukasz.Fojecki@rsyd.dk*](mailto:Grzegorz.Lukasz.Fojecki@rsyd.dk) *Team Station 1*

Appendix 3: Reasons for being seen by an urologist

| **Diagnoses/problems** | n=54 |  |
| --- | --- | --- |
| Postoperative evaluation (rounds) | 12 | 22.2% |
| UTI | 9 | 16.7% |
| Discharge | 5 | 9.3% |
| Urolithiasis | 5 | 9.3% |
| Haematuria | 4 | 7.4% |
| Hydronephrosis | 3 | 5.6% |
| Foley catheter problem | 2 | 3.7% |
| Lymphocele | 2 | 3.7% |
| Polyuria | 2 | 3.7% |
| Preoperative information | 2 | 3.7% |
| Epididymitis | 1 | 1.9% |
| Hematoma | 1 | 1.9% |
| Inadvertently removed suprapubic catheter | 1 | 1.9% |
| Infection after transurethral prostate biopsy | 1 | 1.9% |
| Paraphimosis | 1 | 1.9% |
| Surgical site infection | 1 | 1.9% |
| Suspected torsio testes | 1 | 1.9% |
| Urine retention | 1 | 1.9% |
